# Foveal Avascular Zone and Choroidal Thickness Are Decreased in Subjects with Hard Drusen and without High Genetic Risk of Developing Alzheimer’s Disease

**DOI:** 10.3390/biomedicines9060638

**Published:** 2021-06-02

**Authors:** Inés López-Cuenca, Rosa de Hoz, Celia Alcántara-Rey, Elena Salobrar-García, Lorena Elvira-Hurtado, José A. Fernández-Albarral, Ana Barabash, Federico Ramírez-Toraño, Jaisalmer de Frutos-Lucas, Juan J. Salazar, Ana I. Ramírez, José M. Ramírez

**Affiliations:** 1 Instituto de Investigaciones Oftalmológicas Ramón Castroviejo, Universidad Complutense de Madrid (UCM), IdISSC, 28040 Madrid, Spain; inelopez@ucm.es (I.L.-C.); rdehoz@med.ucm.es (R.d.H.); celiaalc@ucm.es (C.A.-R.); elenasalobrar@med.ucm.es (E.S.-G.); marelvir@ucm.es (L.E.-H.); joseaf08@ucm.es (J.A.F.-A.); jjsalazar@med.ucm.es (J.J.S.); 2 OFTARED—Instituto de Salud Carlos III, 28029 Madrid, Spain; 3 Departamento de Inmunología, Oftalmología y ORL, Facultad de Óptica y Optometría, Universidad Complutense de Madrid, 28037 Madrid, Spain; 4 Endocrinology and Nutrition Department, Instituto de Investigación Sanitaria, Hospital Clínico Universitario San Carlos, 28040 Madrid, Spain; ana.barabash@gmail.com; 5 Centro de Investigación Biomédica en Red de Diabetes y Enfermedades Metabólicas Asociadas, Instituto de Salud Carlos III, 28029 Madrid, Spain; 6 Laboratory of Cognitive and Computational Neuroscience, Center for Biomedical Technology, Technical University of Madrid, 28233 Madrid, Spain; federico.ramirez@ctb.upm.es (F.R.-T.); jaisamer.defrutos@ctb.upm.es (J.d.F.-L.); 7 Department of Experimental Psychology, Universidad Complutense de Madrid, 28223 Madrid, Spain; 8 Centre for Precision Health, Edith Cowan University, Joondalup, WA 6027, Australia; 9 Departamento de Psicología, Facultad de Ciencias de la Vida y de la Naturaleza, Universidad Antonio de Nebrija, 28015 Madrid, Spain; 10 Departamento de Inmunología, Oftalmología y ORL, Facultad de Medicina, Universidad Complutense de Madrid, 28040 Madrid, Spain

**Keywords:** Alzheimer’s, family history, ApoE ɛ4, AMD, choroid, foveal avascular zone, hard drusen, retina, OCT, OCTA

## Abstract

A family history (FH+) of Alzheimer’s disease (AD) and ɛ4 allele of the ApoE gene are the main genetic risk factors for developing AD, whereas ɛ4 allele plays a protective role in age-related macular degeneration. Ocular vascular changes have been reported in both pathologies. We analyzed the choroidal thickness using optical coherence tomography (OCT) and the foveal avascular zone (FAZ) using OCT-angiography and compared the results with ApoE gene expression, AD FH+, and the presence or absence of hard drusen (HD) in 184 cognitively healthy subjects. Choroidal thickness was statistically significantly different in the (FH−, ɛ4−, HD+) group compared with (i) both the (FH−, ɛ4−, HD−) and the (FH+, ɛ4+, HD+) groups in the superior and inferior points at 1500 μm, and (ii) the (FH+, ɛ4−, HD+) group in the superior point at 1500 μm. There were statistically significant differences in the superficial FAZ between the (FH+, ɛ4−, HD+) group and (i) the (FH+, ɛ4−, HD−) group and (ii) the (FH+, ɛ4+, HD−) group. In conclusion, ocular vascular changes are not yet evident in participants with a genetic risk of developing AD.

## 1. Introduction

Alzheimer’s disease (AD) is the most common cause of dementia, responsible for 60–70% of cases [1]. This neurodegenerative disease is characterized by a continuous and irreversible pathological process that begins 15–20 years before the onset of clinical symptoms [2]. The main pathological features are the hyperphosphorylation of the Tau protein and deposition of amyloid β-protein (Aβ) [3], which aggregates in the cerebral vessel walls [4], leading to cerebral amyloid angiopathy (CAA) [5]. These vascular amyloid deposits primarily consist of Aβ_1–40_ and Aβ_1–42_ [6], but *N*-terminal-truncated forms of Aβ and other proteins such as Apolipoprotein E (ApoE) and the α2-macroglobulin receptor/LDL receptor-related protein are also found in these deposits [7,8]. Reduced blood and lymphatic flow [9], the impairment of the gliovascular unit [10], and alterations in both vessel diameter and peripheral immune cell accessibility [11] can result in cerebral vascular deposits and lead to a series of events that result in neurodegeneration [12]. About 85% of AD patients exhibit CAA [13], and it has been reported to be an early and fundamental contributor to the development of the disease and a reliable predictor of cognitive decline [14].

There are similarities between cerebral and retinal vessels [15], and the vascular changes that occur in AD share common pathogenic mechanisms in both tissues [15,16,17]. For this reason, the retinal vascular changes observed in AD can be used to monitor alterations caused by this pathology in the central nervous system. Ocular vascularization has the particularity of being supplied by two different systems, which differ in their regulatory mechanisms and perfusion pressure [18]. While the inner retina is nourished by blood vessels derived from the central retinal artery (CRA), the outer retina is supplied by the choriocapillaris of the choroid [18].

Genetic factors play a critical role in the development of late-onset AD. Two of the most important risk factors are (i) having a first-degree family history of the disease [19] and (ii) being a carrier of at least one ɛ4 allele of ApoE [20]. Children of parents with AD have a six-fold greater risk of developing the disease compared with those without a family history [21].

ApoE is a multi-function protein; it is polymorphic and has three isoforms (ɛ2, ɛ3, and ɛ4). This protein is highly expressed in the liver, brain, and retina [22,23], where the retinal pigmented epithelium (RPE)/choroid complex has significant levels of ApoE mRNA [23]. The ɛ2, ɛ3, and ɛ4 isoforms exhibit differences in lipid binding and confer genetic risks for several diseases of aging, including atherosclerosis, AD, and age-related macular degeneration (AMD) [24]. A single ɛ4 allele increases the risk of developing AD [20], whereas it is associated with a protective effect against AMD [25]. In AD, the ɛ4 allele alters the way that neurons process the amyloid precursor protein (APP) through a cholesterol-mediated pathway [24]. Carriers of two copies of ApoE ɛ4 have shown reduced C-reactive protein (CRP) levels compared with non-carriers, suggesting that the ApoE isoform plays a mediating role in the inflammatory response involved in AMD etiology [26].

In addition, the role of ɛ2, which is protective against AD [20], has been extensively studied in AMD [24,25]. It is associated with a slightly increased risk of developing late AMD, and female ɛ2 carriers have a higher risk of progression compared with female ɛ3 carriers [27].

AMD is a degenerative disorder of the central retina. This pathology has a higher prevalence in patients over 65 years of age and is the main cause of blindness in this age group [28]. In early stages, the pathological changes are characterized by the presence of drusen and changes in the RPE. Drusen are focal deposits composed mainly of extracellular matrix deposits and inflammatory components located between the basal lamina of the RPE and the inner collagenous layer of Bruch’s membrane. The formation of these deposits is due to the continuous phagocytosis and deposition of photoreceptor outer segment components, resulting in an imbalance between the production and clearance of lipid material [29]. AD and AMD share environmental risk factors and histopathological features, particularly the deposition of Aβ in ocular drusen and in senile brain plaques [30]. Analysis of the eyes of aging individuals with AMD by electron microscopy revealed that the basement membranes of retinal capillaries were considerably thicker compared with those of younger individuals. In addition, advanced cases of AMD were associated with a higher proportion of acellular capillaries, which were non-functional, predisposing these patients to ischemia in the inner retina [31]. Changes in the choroid and choroidal microcirculation have been reported in AD [32,33,34] and play an important role in the pathogenesis of AMD [35].

The aim of the present study was to analyze differences in choroidal thickness and the retinal foveal avascular zone (FAZ) and assess whether the findings were associated with ApoE gene expression, AD family history, and the presence or absence of hard drusen in cognitively healthy subjects.

## 2. Materials and Methods

### 2.1. Study Design

This study is part of the project “The cognitive and neurophysiological characteristics of subjects at high risk of developing dementia: a multidimensional approach” (COGDEM study) conducted by the Ramon Castroviejo Institute of Ophthalmic Research (IIORC) of the Complutense University of Madrid (UCM), the Centre for Biomedical Technology (CBT), and the Hospital Clínico San Carlos (HCSC), Madrid, among others. All participants provided written informed consent, and the research followed the tenets of the declaration of Helsinki. This study was approved by the local Ethics Committee (HCSC) with the internal code 18/422-E_BS.

**Figure 1:**
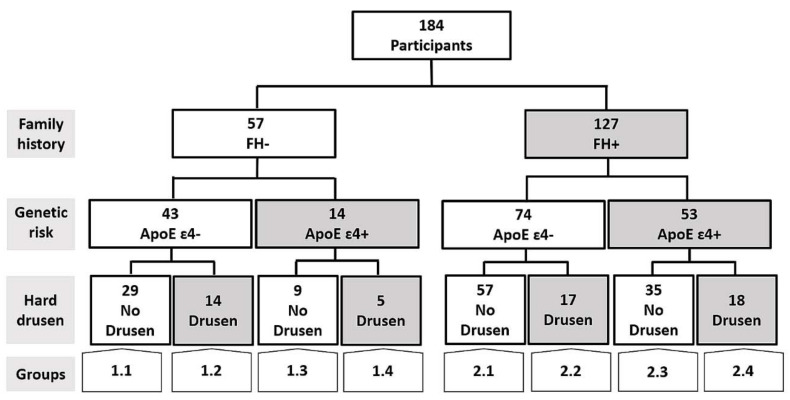
Flow diagram of the patients included in the present work. FH+: subjects with a family history of Alzheimer’s disease (AD); FH−: Subjects without a family history of AD.

The inclusion of patients is summarized in Figure 1. We analyzed two major groups:

- Group 1 is a control group, which consisted of middle-aged subjects without a first-degree family history of AD (FH−).
- Group 2 comprises subjects with a family history of AD (FH+). Subjects were middle-aged with at least one parent with sporadic AD. To verify the AD diagnoses of parents, a review of their medical records was conducted by a multidisciplinary diagnostic consensus panel. Only diagnoses that were made under internationally accepted criteria were included. Because autopsies were not performed in most AD patients, postmortem reports were welcome but were not used as the basis of inclusion. Relatives with known autosomal dominant mutations (i.e., preseniline-1 or 2) were not included.

Both groups were matched in terms of age, socioeconomic status, and other demographic characteristics and had no history of neurological or psychiatric disorders or serious medical conditions. Both groups had normal scores on the Mini-Mental State Examination (MMSE) (above 26) and normal MRIs, with no evidence of brain lesions or pathology.

The two main groups were each subdivided into four subgroups. First, carriers and non-carriers of ApoE ɛ4 were assigned to different groups, which were then subdivided into groups with and without hard drusen in the retina.

The groups are represented as follows:

1.1. FH−; ApoE ɛ4−; No Drusen (FH−, ɛ4−, HD−).
1.2. FH−; ApoE ɛ4−; Drusen (FH−, ɛ4−, HD+).
1.3. FH−; ApoE ɛ4+; No Drusen (FH−, ɛ4+, HD−).
1.4. FH−; ApoE ɛ4+; Drusen (FH−, ɛ4+, HD+).
2.1. FH+; ApoE ɛ4−; No Drusen (FH+, ɛ4−, HD−).
2.2. FH+; ApoE ɛ4−; Drusen (FH+, ɛ4−, HD+).
2.3. FH+; ApoE ɛ4+; No Drusen (FH+, ɛ4+, HD−).
2.4. FH+; ApoE ɛ4+; Drusen (FH+, ɛ4+, HD+).

### 2.2. Subjects

In this prospective study, we included participants from COGDEM’s Database, which consists of 251 subjects. The participants had to be free of ophthalmological pathology, which we confirmed through phone screening.

The participants were examined in the clinic of IIORC. The phone screening questions, visual exams, and inclusion criteria are described in Table 1.

We included 57 and 127 participants with and without a family history of AD, respectively. In addition, we classified participants on the basis of whether they carried the ApoE ɛ4 allele and whether they had hard drusen.

Figure 1 shows a flow diagram that illustrates the different study groups included in the present work.

### 2.3. ApoE Genotyping

Genomic DNA was extracted from whole blood in EDTA using standard DNA isolation methods (DNAzol^®^; Molecular Research Center, Inc., Cincinnati, OH, USA) from FH+ and FH− subjects. Two single-nucleotide polymorphisms (SNPs), rs7412 and rs429358, were genotyped using TaqMan Genotyping Assays on an Applied Biosystems 7500 Fast Real-Time PCR instrument (Applied Biosystems, Forster City, CA, USA). APOE haplotypes were accordingly established. Sample controls for each genotype and negative sample controls were included in each assay. Several intra- and interplate duplicates of DNA samples were included.

### 2.4. Spectral-Domain Optical Coherence Tomography (OCT) Imaging: Choroidal Thickness and FAZ Measurement

**Figure 2:**
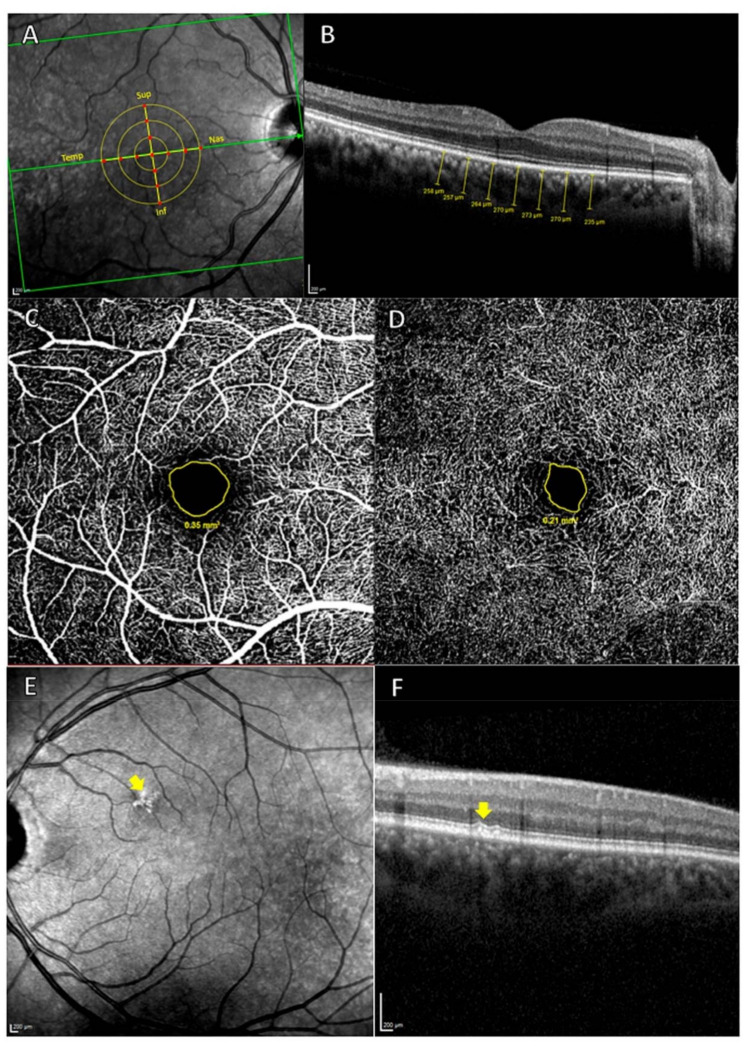
(**A**,**B**) measurement of the choroidal thickness. (**A**) Retinal zone analyzed. The 13 red points indicate where the choroidal thickness measurements were carried out. Sup: superior; Nas: Nasal; Inf: Inferior; Temp: Temporal. (**B**) Choroidal thickness measur measurements (µm). (**C**,**D**), measurement of FAZ. (**C**) OCTA of superficial vascular plexus, with the avascular area outlined in yellow. (**D**) OCTA of deep vascular plexus, with the avascular area outlined in yellow and, (**E**) and (**F**), hard drusen analysis by OCT. (**E**) The yellow arrow shows hyper-reflective shapes in the HRA fundus image. (**F**) Cross-Sectional OCT. The yellow arrow indicates hard drusen localized between the basal lamina of the RPE and the inner collagen layer of Bruch’s membrane.

The choroidal thickness and the foveal avascular zone (FAZ) were measured by OCT Spectralis (Heidelberg Engineering, Heidelberg, Germany). High-quality scans were defined by a minimum signal-to-noise ratio of 25 and an average of 16 B-scans. The choroidal thickness was delimited manually and perpendicularly to the retina by the same examiner using the measurement function in Heidelberg software (Heidelberg, Germany, version 1.10.4.0). The choroidal thickness was measured from the outer hyper-reflective line to the sclerochoroidal interface of the RPE. These measurements were made in the subfoveal choroid and superior, inferior, nasal, and temporal sectors at 500, 1000, and 1500 µm from the center of the fovea (Figure 2A,B).

The Spectralis OCT angiography (OCTA) module was used to measure the superficial and deep FAZ. The avascular area of each plexus was delimited manually with the area measurement tool in Heidelberg software (Figure 2C,D).

Hard drusen were identified as hyper-reflective shapes on high-reflectance acquisition (HRA) fundus images and as hyper-reflective material located between the basal lamina of the RPE and the inner collagen layer of Bruch’s membrane on cross-sectional OCT scans (Figure 2E,F). All OCT scans were analyzed by the same ophthalmologist who determined the type of deposit.

Both the measurements that were performed manually and the classification of the deposits were carried out blindly to avoid the possibility that the information from the participants could influence the measurements carried out by the professionals.

### 2.5. Statistical Analysis

SPSS 25.0 (SPSS Inc., Inc., Chicago, IL, USA) was used to perform the statistical analysis. The differences between study groups were analyzed using the Mann–Whitney test. Data are reported as the median (interquartile range). The chi-square test was used for the analysis of qualitative variables. A *p*-value < 0.05 was considered statistically significant.

## 3. Results

### 3.1. Demographic Data

Eight study groups were included in this work. The demographic data are shown in Table 2.

Groups 1.1, 1.2, 1.3, and 1.4 consisted of individuals without a family history of AD. Group 1.1 (FH−, ɛ4−, HD−) was formed by 29 subjects (12 males) with a mean age of 59.0 (54.0–65.0) and mean MMSE score of 29.0 (28.0–29.0). Group 1.2 (FH−, ɛ4−, HD+) was formed by 14 participants (6 males) with a mean age of 62.5 (56.0–69.0) and mean MMSE score of 29.0 (29.0–29.0). Group 1.3 (FH−, ɛ4+, HD−) had 9 subjects (3 males) with a mean age of 63.0 (54.0–70.0) and mean MMSE score of 29.0 (28.0–30.0), and Group 1.4 (FH−, ɛ4+, HD+) had 5 participants (0 males) with a mean age of 63.0 (58.0–76.5) and mean MMSE score of 29.0 (29.0–30.0).

Groups 2.1, 2.2, 2.3, and 2.4 included individuals with a family history of AD. Group 2.1 (FH+, ɛ4−, HD−) comprised 57 participants (22 males) with a mean age of 58.0 (53.0–62.0) and mean MMSE of 29.0 (28.5–29.0). Group 2.2 (FH+, ɛ4−, HD+) had 17 subjects (6 males) with a mean age of 63.0 (56.5–68.5) and mean MMSE score of 29.0 (28.0–29.0). Group 2.3 (FH+, ɛ4+, HD−) included 35 subjects (11 males) with a mean age of 57.0 (57.0–65.0) and mean MMSE score of 29.0 (29.0–29.0), and Group 2.4 (FH+, ɛ4+, HD+) had 18 subjects (9 males) with a mean age of 55.5 (51.0–63.0) and mean MMSE score of 29.0 (28.0–30.0).

### 3.2. Choroidal Thickness

The choroidal thickness of Group 1.2 (FH−, ɛ4−, HD+) was statistically different (*p* < 0.05) from that of (i) Group 1.1 (FH−, ɛ4−, HD−) in the superior and inferior points at 1500 μm, (ii) Group 2.2 (FH+, ɛ4−, HD+) in the superior point at 1500 μm, and (iii) Group 2.4 (FH+, ɛ4+, HD+) in the superior and inferior points at 1500 μm (Table 3 and Table 4).

### 3.3. Foveal Avascular Zone (FAZ)

There were statistically significant differences in the superficial FAZ between Group 2.1 (FH+, ɛ4−, HD−) (0.51 (0.39–0.62)) and Group 2.2 (FH+, ɛ4−, HD+) (0.67 (0.62–0.80)) (Table 3 and Table 4). In addition, there were significant differences (*p* < 0.05) in the superficial FAZ between Group 2.2 (FH+, ɛ4−, HD+) (0.67 (0.62–0.80)) and Group 2.3 (FH+, ɛ4+, HD−) (0.54 (0.44–0.68)) (Table 3 and Table 4).

## 4. Discussion

The present study demonstrates that ocular vascular changes are not yet evident in participants with a genetic risk of developing AD, while in participants without genetic risk for developing AD who have HD, changes in choroidal thickness are presented. In addition, superficial FAZ also showed small changes between the (FH+, ɛ4−, HD+) group and the (FH+, ɛ4−, HD−) and the (FH+, ɛ4+, HD−) groups.

Changes in the retinal vasculature have been identified as potential biomarkers of AD [36,37,38]. One of the most important ocular vascular layers is the choroid, whose flow is supplied by the posterior ciliary arteries, which are branches of the ophthalmic artery [39]. The choroid has one of the highest blood flows of any tissue in the body, and its primary function is to provide nutrients and oxygen to the outer retina, including the RPE and photoreceptors [40,41]. The choroidal circulation is controlled mainly by autonomic and sensory innervation and not by a self-regulatory mechanism [39,42]. Nerve fibers that regulate choroidal vascularization are predominantly located in the submacular region, where most NPY+ and TH+ ganglion cells are also concentrated [43]. This neuronal distribution in the submacular region suggests the possibility that vascular conditions of certain eye diseases, such as diabetic macular edema or AMD, may be related to the dysfunction of these cells [44]. Some authors have correlated AD with AMD, so it is not surprising that choroidal vascular changes appear before retinal changes in AD [30].

A decrease in choroidal thickness has been reported in patients with AD compared with healthy older subjects [15,33,34,45,46,47,48,49]. These changes have also been observed in early stages of the disease [32], including in patients with preclinical and prodromal AD [49] and those with mild cognitive impairment (MCI) [41]. Choroidal thinning may indicate abnormal choroidal blood supply associated with hypoperfusion or atrophic changes related to various pathological events, with cerebral Aβ accumulation being the main trigger [33]. Capillary occlusion detected in imaging studies provides a possible explanation for hypoperfusion in AD brains [50,51].

In the present study, no differences were observed between groups with a high genetic risk of developing AD (subjects with a family history and carriers of ApoE ɛ4) and control groups (participants without a family history and non-carriers of ApoE ɛ4). One possible explanation is that our participants are cognitively healthy people, and it is unknown whether they will develop the disease in the future. Although genetic factors play an important role in determining a person’s risk of developing the disease [52], there are other contributing factors, such as the influence of the environment or modifiable risk factors, including physical activity, diet, and alcohol consumption, all of which can modify the course or even the onset of the disease [53].

The statistically significant differences found in our study are in groups that have hard drusen. In addition, the thinnest choroids correspond to subjects who have no family history of the disease and are non-carriers of ApoE ɛ4 (Group 1.2; FH−, ɛ4−, HD+). ApoE ɛ4 is known to be a protective factor for AMD, and the risk of late (end-stage) AMD in individuals of Caucasian descent is 20–50% lower than that of carriers of the ɛ3 allele [54,55], while the ɛ2 allele is associated with increased disease progression in women [27]. However, the association between ApoE ɛ4 and protection is stronger than that between ɛ2 and risk [25].

The allelic variants of the ApoE gene represent one of the most important genetic risk factors for developing AMD [55]. ApoE plays a role in cell-membrane remodeling and is essential for the normal function and maintenance of the retina [54]. Lipid transport across Bruch’s membrane is easier in carriers of the ɛ4 allele compared with ɛ2 and ɛ3 allele carriers. The positive charges in proteins coded by ɛ4 alleles account for their improved ability to clear debris because they interact with the hydrophobic barrier generated by the accumulation of neutral lipids [56].

The formation of drusen is not random but is influenced by the anatomy of the choroid, and the mechanisms leading to the formation of these deposits in intercapillary areas or areas devoid of capillary lumens are unknown [57]. Drusen are associated with decreased choriocapillaris density and decreased choroid flow [57,58,59]. Similarly, reduced blood flow to the choriocapillaris can lead to dysfunction of the RPE, promoting further accumulation of debris in the form of drusen or basal lamellar deposits [58]. In early AMD, choroidal thickness already tends to be thinner than in normal eyes [60]. The exact cause of this thinning is unknown; it may be a response to choroidal atrophy or hypoxia, or it may be a secondary response to the accumulation of deposits or damage to the RPE [60].

Numerous studies with different imaging techniques have shown choroidal changes in AMD, but no consensus has been reached. Previous studies with fluorescein angiography have reported abnormalities in choroidal perfusion, such as decreased blood flow, increased fluorescein blockage [61,62], and areas of delayed choroidal perfusion, which were associated with decreased visual function [63]. In another study, slow choroidal filling on fluorescein angiography was reported to be a significant risk factor for developing geographic RPE atrophy, suggesting the importance of ischemia in this etiology [64]. In addition, in another study in subjects with dry AMD, an increase in arterial filling time was found, suggesting a decrease in choroidal blood flow [65].

In a recent study using color Doppler imaging, AMD patients showed decreased blood velocity and increased pulsatility in the central retinal artery and posterior short ciliary arteries [66]. In other neurodegenerative diseases, such as AD or previous stages such as MCI, the accumulation of vascular deposits in the retina (Aβ_40_ and Aβ_42_) is also associated with vascular changes [67]. The accumulation of Aβ was shown to reduce the expression of LDL receptor-related protein-1 (LRPG), leading to a decrease in the expression of vascular platelet-derived growth factor receptor-β (PDGFRβ) and an increase in pericyte death by apoptosis [12]. Brain pericytes and vascular smooth muscle cells are critical in regulating blood flow and the integrity of the blood–brain barrier [68]. Because of its similarity to the blood-retinal barrier, damage at this level is implicated in pathologies such as AMD and diabetic retinopathy [12]. Although drusen are one of the first signs to appear in AMD, the subjects in our study do not have AMD, so we cannot claim that the changes in choroidal thickness are comparable to those found in patients with AMD. The vascular changes in the choroid in our patients may be a very early sign secondary to altered blood flow.

On the other hand, retinal circulation is characterized by low blood flow, high perfusion pressure [39], and three distinct structures: radial peripapillary capillaries, superficial vascular, and deep vascular plexus [15]. In the foveal zone, in both the deep and superficial plexus, there is a capillary-free zone called the FAZ [69]. The enlargement of the FAZ is a sign of ischemia and is detected in cases of diabetic retinopathy and macular vein branch occlusion [70]. This parameter has received interest as a biomarker for the monitoring and follow-up of pathologies such as AD [15]. For this reason, the FAZ has been studied at different stages of the disease using OCTA.

In our study, among the participants without drusen, we found no statistically significant differences between those with a high genetic risk of AD (group 2.3) and those without a genetic risk of AD (group 1.1). Our results are similar to those of a previous study in preclinical AD, in which no differences in the FAZ were found between Aβ+ patients and controls (Aβ−), who had lower vascular density [71]. However, in a previous study, the FAZ of cognitively healthy individuals who had preclinical AD and positive biomarkers for AD, such as PET scanning for PiB or ^18^F-AV-45 and Aβ_42_+ levels in the cerebrospinal fluid (CSF), was increased in comparison with participants without these biomarkers. However, information about the family histories of the participants and results of genetic testing (ApoE ɛ4 status) were not collected [72]. In our analysis of the FAZ, the only statistically significant differences were in the superficial FAZ in Group 2.2 (FH+, ɛ4−, HD+) in comparison with both Groups 2.1 (FH+, ɛ4−, HD−) and 2.3 (FH+, ɛ4+, HD−). These changes are consistent with those found in studies of patients with MCI, who showed a decrease in vascular density and, therefore, an increase in the FAZ area [38,73,74]. In a recent study in patients with MCI, an increased deep FAZ was observed compared with controls. The same study analyzed the influence of ApoE ɛ4 on vascularization in people with and without MCI and found no significant differences [75].

The results obtained in FAZ analyses in AD patients are quite diverse. Some studies have found no statistically significant differences between the FAZ of AD patients and healthy controls [32,76]. However, others have found differences in both the superficial [15,77] and deep FAZ [78] compared with controls. An increased FAZ is attributed to reduced angiogenesis, a consequence of decreased vascular endothelial growth factor (VEGF), which binds to Aβ protein plaques [15], as well as the competitive binding of Aβ to the VEGF-2 receptor [79,80].

These discrepancies in the results of different studies may be explained by the degree of cognitive impairment of the participants. However, our participants were cognitively healthy, so the increased area of the superficial FAZ could reflect compromised blood perfusion, which may trigger the deposition of drusenoid material.

One of the strengths of this study is the strict selection of participants. Only young, cognitively healthy participants with a family history of Alzheimer’s disease were included in the study. Another strength is the knowledge of the genetic characterization of the subjects. However, among the main limitations of the study are the small sample size in some of the study groups (there are groups with only 9 and 5 participants) and its retrospective character. Longitudinal studies will be necessary to know the evolution of all participants.

## 5. Conclusions

In conclusion, in this study, ocular vascular changes are not yet evident in healthy cognitive participants at high genetic risk of developing AD. The statistically significant differences in our study are in subjects who have hard drusen who should be evaluated periodically. In addition, the thinnest choroids correspond to subjects who have no family history of AD and are non-carriers of ApoE ɛ4.

## Figures and Tables

**Table 1:** Ophthalmological evaluation of COGDEM participants.

Ophthalmological Evaluation		
Screening Questions	Visual Exam	Inclusion Criteria
Do you use glasses? Yes/no Do you know if you have myopia, hypermetropy, or astigmatism? Yes/no Do you know your diopter measurements? Yes/no Do you have any ocular pathologies? Yes/no Do you receive any type of ocular treatment? Yes/no Have you undergone any type of ocular surgery? Yes/no	Refraction Visual acuity Biomicroscopy Intraocular pressure OCT/OCTA	±5 Spherocylindrical refractive >0.5 dec <21 mmHg Free of ocular disease Free of congenital malformation Free of known or suspected glaucoma

OCT: optical coherence tomography; OCTA: OCT angiography; dec: decimal scale.

**Table 2:** Demographic data on the participants in the different study groups.

Demographic/MMSE Data	FH−				FH+			
	ApoE ɛ4−		ApoE ɛ4+		ApoE ɛ4−		ApoE ɛ4+	
	No HD	HD	No HD	HD	No HD	HD	No HD	HD
	Group 1.1	Group 1.2	Group 1.3	Group 1.4	Group 2.1	Group 2.2	Group 2.3	Group 2.4
***N***	29	14	9	5	57	17	35	18
**Age**	59.0 (54.0–65.0)	62.5 (56.0–69.0)	63.0 (54.0–70.0)	63.0 (58.0–76.5)	58.0 (53.0–62,0)	63.0 (56.5–58.5)	57.0 (57.0–65.0)	55.5 (51.0–63.0)
**Sex Male/Female**	12/17	6/8	3/6	0/5	22/35	6/11	11/24	9/9
**MMSE**	29.0 (28.0–29.0)	29.0 (29.0–29.0)	29.0 (28.0–30.0)	29.0 (29.0–30.0)	29.0 (28.5–29.0)	29.0 (28.0–29.0)	29.0 (29.0–29.0)	29.0 (28.0–30.0)

Median (interquartile range); FH+, subjects with a family history of Alzheimer’s disease (AD); FH−, subjects without a family history of AD; ApoE, Apolipoprotein E.

**Table 3:** Median and interquartile range of the FAZ and choroidal thickness in the study groups.

Vascular Areas Analyzed			FH−				FH+			
			ApoE ɛ4−		ApoE ɛ4+		ApoE ɛ4−		ApoE ɛ4+	
			No HD	HD	No HD	HD	No HD	HD	No HD	HD
			Group 1.1	Group 1.2	Group 1.3	Group 1.4	Group 2.1	Group 2.2	Group 2.3	Group 2.4
**FAZ**		**Superficial**	0.47 (0.39–0.62)	0.45 (0.42–0.68)	0.47(0.37–0.84)	0.47 (0.37–0.82)	0.51 (0.39–0.62)	0.67 (0.62–0.80)	0.54 (0.44–0.68)	0.59 (0.43–0.83)
		**Deep**	0.23 (0.18–0.31)	0.22 (0.16–0.34)	0.24 (0.2–0.359	0.23 (0.12–0.34)	0.26 (0.39–0.33)	0.29 (0.24–0.38)	0.28 (0.23–0.33)	0.29 (0.21–0.41)
**Choroidal Thickness**	**Subfoveal**		268.0 (213.5–307.5)	249.5 (176.8–268.0)	257.0 (234.0–318.0)	252.5 (164.5–279.8)	273.0 (231.0–309.5)	255.0 (215.5–287.0)	263.0 (212.0–302.0)	241.0 (224.5–296.5)
	**Temporal**	**500 μm**	260.0 (210.5–325.0)	234.0 (159.8–259.0)	266.0 (231.5–316.0)	256.5 (173.3–296.3)	263.0 (220.5–302.0)	252.0 (199.5–292.0)	256.0 (228.0–295.0)	252.0 (222.8–297.3)
		**1000 μm**	248.0 (207.5–316.0)	219.5 (161.8–267.50)	276.0 (202.5–300.0)	256.5 (176.5–310.3)	263.0 (218.5–288.5)	248.0 (195.5–292.0)	253.0 (219.0–287.0)	247.5 (217.5–282.0)
		**1500 μm**	232.0 (202.0–296.0)	222.5 (157.5–255.3)	254,0 (186.5–343.0)	255.0 (189.8–292.5)	255.0 (216.0–288.5)	251.0 (202.5–282.0)	250.0 (215.0–280.0)	265.0 (214.5–291.0)
	**Nasal**	**500 μm**	261.0 (204.5–313.5)	230.5 (177.0–252.5)	238.0 (228.0–302.5)	256.5 (180.3–292.3)	267.0 (220.5–295.0)	234.0 (198.5–285.5)	249.0 (199.0–297.0)	244.0 (224.0–286.2)
		**1000 μm**	233.0 (195.5–303.5)	206.0 (150.8–282.0)	223.0 (204.5–291.5)	257.0 (173.3–295.8)	255.0 (190.5–299.0)	225.0 (191.0–274.5)	240.0 (182.0–281.0)	248.5 (198.8–268.0)
		**1500 μm**	221 (175.50–278.5)	182.5 (140.5–269.3)	217.0 (171.0–278.0)	271.0 (134.0–300.0)	228.0 (183.5–274.5)	205.0 (170.5–242.0)	218.0 (174.0–265.0)	252.0 (179.8–263.0)
	**Superior**	**500 μm**	262.0 (218.0–301.0)	222.0 (170.8–273.5)	260.0 (228.5–311.5)	265.5 (182.8–292.8)	265.0 (228.0–299−0)	262.0 (219.5–282.5)	257.0 (209.0–309.0)	252.5 (222.0–284.0)
		**1000 μm**	255.0 (218.5–297.0)	227.0 (170.8–263.8)	256.0 (227.5–308.5)	272.5 (180.8–281.8)	271.0 (228.5–302.0)	259.0 (217.5–289.0)	267.0 (223.0–299.0)	252.5 (234.3–288.0)
		**1500 μm**	259.0 (209.5–306.5)	223.0 (147.0–253.3)	250.0 (225.5–319.0)	269.0 (174.0–298.0)	261.0 (224.5–305.5)	261.0 (206.0–286.0)	262.0 (226.0–300.0)	257.5 (233.3–290.3)
	**Inferior**	**500 μm**	262.0 (209.5–311.5)	222.5 (172.8–274.3)	256.0 (222.0–308.5)	272.0 (179.5–284.3)	270.0 (231.5–304.0)	252.0 (208.5–276.5)	265.0 (216.0–310.0)	251.5 (216,3–301,3)
		**1000 μm**	266.0 (217.0–306.5)	219.0 (165.0–270.8)	267.0 (229.5–308.5)	267.5 (171.0–296.5)	271.0 (227.5–295.5)	252.0 (211.5–268.0)	270.0 (216.0–322.0)	243.0 (219.8–287.3)
		**1500 μm**	264.0 (213.5–312.0)	217.0 (169.0–257.25)	262.0 (218.5–300.5)	271.5 (183.0–276.0)	268.0 (224.0–304.0)	257.0 (211.0–266.5)	250.0 (214.0–317.0)	269.0 (216.0–309.5)

Median (interquartile range); FH+, subjects with a family history of Alzheimer’s disease (AD); FH, subjects without a family history of AD; HD, hard drusen; ApoE, Apolipoprotein E; FAZ, Foveal avascular zone.

**Table 4:** Significant *p*−values for differences between groups in the FAZ and choroidal thickness. *p*−values are in parentheses.

Study Groups				FH−				FH+			
				ApoE ɛ4−		ApoE ɛ4+		ApoE ɛ4−		ApoE ɛ4+	
				No HD	HD	No HD	HD	No HD	HD	No HD	HD
				Group 1.1	Group 1.2	Group 1.3	Group 1.4	Group 2.1	Group 2.2	Group 2.3	Group 2.4
**FH−**	**ApoE** **ɛ** **4−**	No HD	1.1		Choroid: Sup 1500 (0.030), Inf 1500 (0.028)						
		HD	1.2						Choroid: Sup 1500 (0.039)		Choroid: Sup 1500 (0.019), Inf 1500 (0.040)
	**ApoE** **ɛ** **4+**	No HD	1.3								
		HD	1.4								
**FH+**	**ApoE** **ɛ** **4−**	No HD	2.1						Superficial FAZ (<0.001)		
		HD	2.2							Superficial FAZ (0.013)	
	**ApoE** **ɛ** **4+**	No HD	2.3								
		HD	2.4								

*p*-values are in parentheses. FH+, subjects with a family history of Alzheimer’s disease (AD); FH−, subjects without a family history of AD; HD, hard drusen; ApoE, Apolipoprotein E; FAZ, Foveal avascular zone.

## Data Availability

The data supporting the findings of this study are available from the corresponding author upon request.
